# Topical Application of TAT-Superoxide Dismutase in Acupoints LI 20 on Allergic Rhinitis

**DOI:** 10.1155/2016/3830273

**Published:** 2016-12-29

**Authors:** Jing-Ke Guo, Ming-Ming Xu, Mei-Feng Zheng, Shu-Tao Liu, Jian-Wu Zhou, Li-Jing Ke, Tian-Bao Chen, Ping-Fan Rao

**Affiliations:** ^1^SIBS, CAS-Zhejiang Gongshang University Joint Centre for Food and Nutrition Research, Zhejiang Gongshang University, Hangzhou 310018, China; ^2^Acupuncture College, Fujian University of Traditional Chinese Medicine, Fuzhou 350003, China; ^3^College of Biological Science and Technology, Fuzhou University, Fuzhou 350108, China; ^4^Natural Drug Discovery Group, School of Pharmacy, Queen's University, Belfast BT9 7BL, UK

## Abstract

Reactive oxygen species are products of cellular metabolism and assigned important roles in biomedical science as deleterious factors in pathologies. In fact, some studies have shown that the therapeutic benefits of taking antioxidants were limited and the potential for therapeutic intervention remains unclear. New evidences showed that ROS have some ability of intercellular transportation. For treating allergic rhinitis, as a novel intracellular superoxide quencher, TAT-SOD applied to acupoints LI 20 instead of directly to nasal cavity can be used to test that. TTA group apply TAT-SOD cream prepared by adding purified TAT-SOD to the vehicle cream to acupoints LI 20, while placebo group used the vehicle cream instead. TTN group applied the same TAT-SOD cream directly to nasal cavity three times daily. Symptom scores were recorded at baseline and days 8 and 15. For the overall efficacy rate, TTA group was 81.0%, while placebo group was 5.9% and TTN was 0%. Malondialdehyde levels decreased observably in TTA group, and superoxide dismutase, catalase, and glutathione peroxidase levels remained basically unaffected. Enzymatic scavenging of the intracellular superoxide at acupoints LI 20 proved to be effective in treating allergic rhinitis, while no improvement was observed with the placebo group and TTN group.

## 1. Introduction

Reactive oxygen species (ROS) are products of normal cellular metabolism [1, 2] and increasingly assigned important roles in biomedical science as deleterious factors in pathologies and aging [3].

ROS are known to act as second messengers in the intracellular signaling pathways involved in activation of proinflammatory responses [4, 5]. In addition, ROS are widely recognized as important mediators of cell growth, adhesion, differentiation, senescence, and apoptosis [6].

ROS participate not only in intracellular but also intercellular signaling processes [7]. ROS could be released from the mitochondria through voltage dependent anion channels [8]. ROS has been associated with both increased and decreased intercellular transport via plasmodesmata [9]. ROS play important role in mitochondria dependent apoptotic signaling via gap junctional intercellular communication [10]. ROS have some ability of intercellular transportation. Tamas Dolowschiak et al. indicate an important role of ROS in horizontal epithelial cell-cell communication [11]. Wibke Bechtel observed modulation of intercellular ROS signaling of human tumor cells [12].

Understanding such complexity of the ROS signaling is critically hinged on the ability to visualize and quantify local, compartmental, and global ROS dynamics at high selectivity, sensitivity, and spatiotemporal resolution. Many techniques were improved to ROS imaging at levels of intact cells, whole organs or tissues, and even live organisms [13]. In our previous work, reproducible fluorescence lines superimposable to meridian lines were revealed on the frontal interior abdominal wall when intracellular ROS indicators were injected into living SD-rats tail vein, confirming the substantial implication of ROS in meridian system [14]. A controlled study has shown that topical application of superoxide dismutase (SOD) fused with the TAT peptide (TAT-SOD), to various acupoints along the meridian lines used in acupuncture to treat obesity, leads to significant weight loss [15], monitoring the effects of acupoint antioxidant intervention by measuring electrical potential difference along the meridian [16].

Allergic rhinitis (AR) is a global health problem that causes major illness and disability worldwide, including in China [17]. There is increasing evidence that ROS is extensively involved in multiple stages of AR pathogenesis [18]. Theoretically, various antioxidants can be useful in AR treatment [19]. In fact, some studies have shown that the therapeutic benefits of taking antioxidants were limited and the potential for therapeutic intervention remains unclear [20]. Furthermore, as the most primary antioxidant enzyme, SOD, is found to fail to attenuate allergen-induced nasal congestion in ragweed-sensitized dogs by either topical or systemic application [21]. It is obvious that implications of ROS in AR or effective applications of antioxidants to intervene the processes are more complicated than what is understood.

TAT-SOD is a recombinant protein of human liver Cu,Zn-SOD fused with TAT peptide, an 11 peptide derived from HIV-1 transactivator of transcription protein [22], which enables the fusion protein to be delivered cross the cell membrane while maintaining SOD activities, making it an intracellular superoxide quenching enzyme [23]. In the investigation on TAT-SOD's effects on human facial skin, we accidentally discovered that smearing of TAT-SOD containing cream on certain facial acupoints (acupoints LI 20) could mitigate AR symptoms such as itching, congestion, and sneezing. What is worth noting is that topical TAT-SOD needed to be applied to the acupoints LI 20 to be effective and applications either to nonacupoint areas or even directly to the inflammatory nasal mucosa could not improve AR symptom.

Acupoints are locations on the body for needle stimulation in acupuncture, which is believed to achieve therapeutic effects [24]. Acupuncture is extensively used to treat AR employing such acupoints including indispensably acupoints LI 20. Meanwhile, there is a considerable increase in public acceptance of acupuncture as well as the number of rigorously controlled trials [25]. The association between ROS and acupuncture meridians may provide useful insight into the mechanism of effects of the removal of the intracellular ROS in acupoints and intercellular transportation of ROS on AR symptoms. This study aims to validate our previous discovery by a double-blind and placebo-controlled study, including blood test for antioxidant status, in hope to further elucidate complicated roles of ROS.

## 2. Materials and Methods

### 2.1. Setting

This study was performed with central enrollment and allocation by Hospital of Fujian Traditional Chinese Medicine University (research center). AR patients between the ages of 18 and 43 years were recruited from October 3, 2012, to August 14, 2013. Written informed consent was obtained from all patients before enrollment and all patients were free to withdraw from the study at any time. The Medical Research Ethics Committee and Institutional Review Board of Fujian Institute of Traditional Chinese Medicine provided approval for this study, and all participants gave informed consent for publication of these data.

### 2.2. Patients

Patients were recruited from physician offices in the research center. These patients had an initial screening with an allergy questionnaire and skin puncture testing to confirm an allergic response to a perennial allergen (cat, dog, dust mite, and indoor mold). The AR patients with a positive skin puncture test and a total nasal symptom score of 5 or greater (of total of 12) entered into the study. The following patients were excluded from this study: patients with a history or physical examination suggestive of renal, hepatic, or cardiovascular disease; pregnant or lactating women; being treated with systemic or topical corticosteroids during the previous 30 days; being treated with oral antihistamines or decongestants during the previous 7 days; being treated with immunotherapy; patients on chronic antiasthma medications; patients with nasal polyps or a significantly deviated septum; patients with a history of an upper respiratory infection within 14 days of study entry. No other medication or treatment was allowed during the study period [26].

### 2.3. Study Schedule

AR patients underwent observation of nasal mucosa before starting the study. At the baseline visit, the physician recorded AR patients' age, sex, and course of disease and scored nasal symptoms before the morning dose. All participants returned to the research center on day 8 for replacement of medications, scoring nasal symptoms, and performance of nasal mucosa observation. On day 15, all participants stopped treatment, returned medication, and performed a final nasal mucosa observation. Adverse events were recorded.

All participants' venous blood samples for biochemistry test were obtained by venepuncture with vacuum tubes on day 0 and day 15 of the treatment period before the morning dose.

### 2.4. Treatment Protocol

After the initial screening, qualified AR patients were randomized into 1 of 3 treatment groups. TTA group were instructed first to locate acupoints and apply 0.1 ml of 5000 U/ml TAT-SOD cream prepared by adding purified TAT-SOD to the vehicle cream in an area of 1 cm^2^ to bilateral acupoint LI 20 [27], which they then conducted at home three times daily, while placebo group used the vehicle cream instead. TTN group applied the same TAT-SOD cream directly to nasal cavity three times daily.

TAT-SOD was prepared by expression of recombinant fusion protein of human Cu,Zn-SOD fused with TAT peptide in* E. coli* and isolated by affinity chromatography to electrophoretically pure for use. TAT-SOD cream was prepared by the homogenization of the isolated TAT-SOD with the vehicle cream (Johnson & Johnson, Shanghai, China) as previously reported. TAT-SOD in the cream is remarkably stable. SOD activity loss was less than 5.6% when TAT-SOD was stored at room temperature for 6 months [15].

Rhinoscopy was performed to observe the color of inferior turbinate mucosa and to determine whether the inferior turbinate swelled, whether the middle turbinate was visible and whether polypoid changes existed in the middle turbinate.

### 2.5. Randomization

The investigator assigned a computer-generated random number to each participant. The investigator was with no clinical involvement in the trial.

### 2.6. Blinding

Both the patients and researchers conducting the study were blinded. The study intervention products were prepared centrally by the hospital pharmacy at the Hospital of Fujian Traditional Chinese Medicine University by independent pharmacist not involved in the conduct of the trial. The active product and placebo were packaged in identical glass jars and labeled with participant code numbers. The appearance and texture of the products were identical. This procedure was performed by an independent pharmacist, who was the only person aware of the codes' meaning. The participant code number's meaning was concealed until all data were analyzed.

### 2.7. Primary Outcome

The main outcome measures were nasal symptoms, consisting of nasal itching, congestion, rhinorrhea, and sneezing. The severity of each nasal symptom recorded on a scale of 0 to 3 (0 = none, 1 = mild, 2 = moderate, and 3 = severe), evaluated by physician on day 0, day 8, and day 15 of the treatment period according to the principles of diagnosis and treatment of AR [28]. We used the percentage improvement (PI) as a secondary efficacy outcome measure, which was calculated with the following formula: PI = (baseline scores − day 15 scores)/baseline scores. The overall clinical effective rate on symptoms was evaluated as deterioration (PI < 0), no improvement (0 ≤ PI < 0.25), moderate (0.25 ≤ PI < 0.65), or marked improvement (PI ≥ 0.65).

AR patients' serum malondialdehyde (MDA) content and SOD, catalase (CAT), and glutathione peroxidase (GPx) activity were analyzed with colorimetric assay kits (Nanjing Jiancheng Bioengineering Institute, Nanjing, China).

### 2.8. Statistical Analysis

The study design called for at least 45 evaluable patients, for each group at least 15 evaluable patients. Ultimately, 56 AR patients were enrolled, with 21 in TTA group, 17 in placebo group, and 18 in TTN group. Sample size estimations were not performed. Because of the complex design of this pilot study, sample size was chosen on the basis of practical considerations. Therefore, this study was not designed to have sufficient power, and the results of statistical testing have to be interpreted as descriptive, explorative, and hypothesis generating rather than as confirmatory. Data are reported as means (SEM). The statistical analysis was performed with SPSS 15.0 (SPSS, Inc., Chicago, IL) and R version 2.9.0 software. Symptom scores and blood biochemistry test results in TTA group and placebo group, TTA group, and TTN group were compared using two-sample *t*-test.

## 3. Results

**Figure 1:**
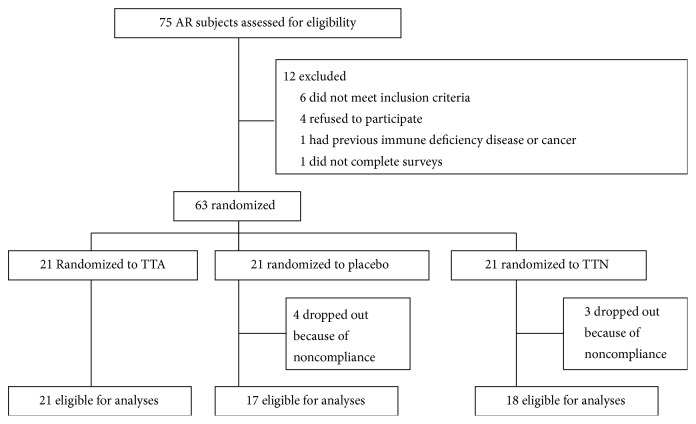
Study flow chart.

We screened 75 subjects and entered 63 subjects into the study. 7 subjects dropped out, and 56 subjects completed the study. The study flow is presented in Figure 1. The groups were matched at entry for age, sex, skin test sensitivity, and course of disease (see Table 1). No adverse events occurred.

The mean rhinitis symptom scores at day 0, day 8, and day 15 were shown in Table 1. After 2-week treatments, the symptom score (total) was decreased from 6.9 to 3.0 with TTA group, from 7.1 to 6.7 with the placebo group, and from 7.1 to 6.9 with TTN group. Of all the three treatments, only topical application of TAT-SOD to acupoints LI 20 significantly improved AR symptom. For the overall efficacy rate, TTA group was 81.0% (17/21), while placebo group was 5.9% (1/17) and TTN was 0% (0/18). In rhinoscopy observation, most patients' inferior turbinate swelled and appeared gray in color with excessive mucus secretion before the treatment. After 2-week treatments, 11 patients' nasal edema disappeared completely in TTA group, while no improvement was observed with the placebo group and TTN group.

**Figure 2:**
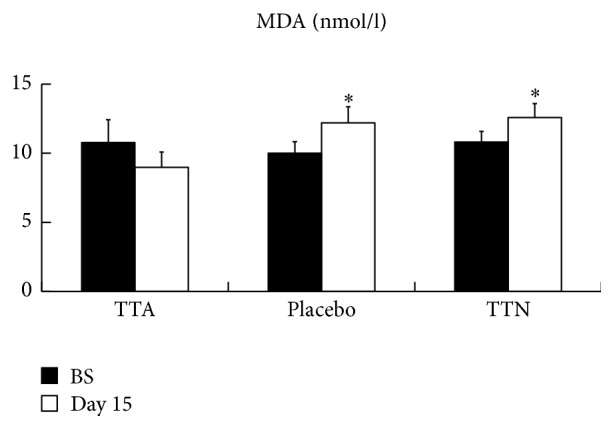
Allergic rhinitis patients' serum malondialdehyde (MDA) content were analyzed. Bars represent the mean ± standard error of the mean. Asterisks denote *p* < 0.05 considered significant compared to TTA group.

**Figure 3:**
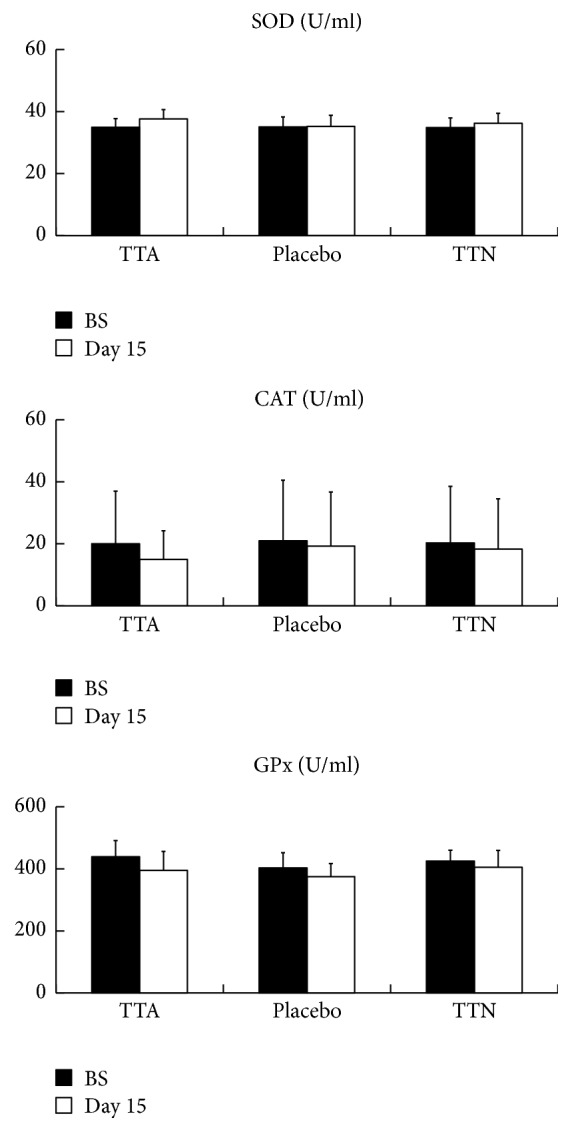
Allergic rhinitis patients' serum SOD, catalase (CAT), and glutathione peroxidase (GPx) activity were analyzed. Bars represent the mean ± standard error of the mean. No significant changes in the serum activities of SOD, CAT, and GPx were observed for all the three groups.

Influences of the treatments on MDA levels are shown in Figure 2. The serum MDA levels with TTA group were decreased from 10.8 nmol/l to 9.0 nmol/l, while those with two other groups were increased. No significant changes in the serum activities of SOD, CAT, and GPx were observed for all the three groups before and after the trial, as is shown in Figure 3.

## 4. Discussion

In this study, enzymatic scavenging of the intracellular superoxide at acupoints was demonstrated to remarkably alleviate AR symptoms. Of all 21 patients received acupoints TAT-SOD treatment, 33.3% (7/21) demonstrated marked improvement and 47.6% (10/21) moderate improvement. In contrast, only 5.9% of patients in the placebo group reported moderate improvement. Those results were also supported by our rhinoscopy observation. The overall efficacy rate of the treatment was 81.0% (17/21), which is higher than 60.0% (6/10) of Astemizole and 67.0% (8/12) of Tefenadine [29] and comparable to the most optimum results of acupuncture treatment [30].

As shown in Table 1, the mean total nasal symptoms score of acupoints LI 20 TAT-SOD treated patients decreased by 3.9 from baseline on day 15 by comparison with a decrease of 0.4 in the same period in placebo group and 0.2 in TTN group.

On each nasal symptom, nasal itching and sneezing represent main characteristic symptoms besides nasal obstruction and rhinorrhea in AR [31]. As shown in Table 1, the itching symptom was significantly alleviated on day 8, followed by the remarkable relief of the sneezing symptom on day 15 of among all nasal symptoms. The sequence of the symptom improvement is the same as treatments with mometasone furoate nasal spray reported by Meltzer et al. [32]. However, no improvement was observed with applying the TAT-SOD cream directly to nasal cavity.

MDA is a marker for oxidative stress [33]. As a good measurement of lipid peroxidation which is one of the chief mechanisms of cell damage [34], MDA level provides another important biomarker reflecting AR progression [35]. In this study, AR patients treated with TAT-SOD cream by topical application at acupoints instead of directly drop into nasal cavity demonstrated a decrease in their serum MDA level from 10.8 nmol/l to 9.0 nmol/l, while the placebo group increased from 10.0 nmol/l to 12.2 nmol/l and the TTN group increased from 10.8 nmol/l to 12.6 nmol/l, indicating smearing TAT-SOD cream at acupoints reduced the nasal epithelial oxidative damage, leading to alleviation of the symptoms.

Histamine is a biogenic amine that plays an essential role in controlling many physiological functions, both in the central nervous system (CNS) and in the peripheral nervous system (PNS) [36]. The effects of histamine have a strong connection with the production of reactive oxygen species [37]. The swelling of nasal mucosa is known to be caused by histamine released from mast cells whose activation is related to a high level intracellular superoxide [18], which is nothing but the very substrate of TAT-SOD. Topical TAT-SOD's effectiveness must have been achieved via the modulation of the intracellular superoxide level of mast cells. Directly applying TAT-SOD to the nasal mucosa failed to improve AR symptom possibly because TAT-SOD was blocked by the swollen tissue from reaching mast cells. The only explanation why TAT-SOD should be applied to distant acupoints to take effect is that a superoxide channel exists between the acupoints and mast cells. It is true that TAT-SOD did not influence the whole system since serum contents of antioxidant enzymes remained unchanged, as is shown in Figure 3. Our results indicate that the effect of smearing TAT-SOD cream at acupoints did not work to boost the general serum antioxidant capability of the patients but to scavenge the intracellular superoxide locally at the acupoints which must have been somehow connected to nasal epithelium, possibly through a meridian line, leading to a lower level of nasal epithelial damage. In acupuncture, it is known that a channel exists between acupoints and the targeted tissue or organ.

As is shown by our previous work [14], these channels may be the alignment of cells with a high content of superoxide, it is possibly a novel channel for superoxide transporting and disposal between acupoints and the action target of acupuncture, as is in this case a channel between LI 20 and nasal epithelium.

**Figure 4:**
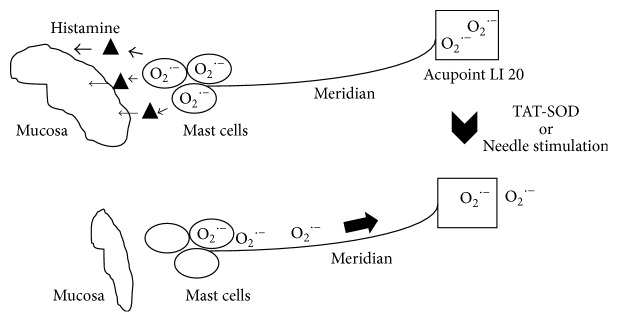
Model of scavenging the intracellular superoxide locally at the acupoints leading to a lower level of nasal epithelial damage through a meridian line. In allergic rhinitis, house dust exposure induces nasal epithelium to produce ROS, which can challenge mast cells to release the proinflammatory mediators, including histamine. Histamine plays a crucial role in the development of nasal blockage, pruritus, sneezing, and rhinorrhea. ROS could control excitability of connective tissues through redox modulation of membrane generating ROS and vice versa. ROS produced in this compartment play a critical role in inflammatory signaling in AR. Meridian lines connecting acupoints and nasal epithelium must be a channel implicated with ROS. Scavenging the intracellular ROS locally at the acupoint leads to lower level of nasal epithelial damage through a meridian line.

As is illustrated in Figure 4, a channel is hypothesized through which superoxide can travel from mast cells to acupoints because of the electric potential change due to intracellular superoxide removal in the acupoints. The channel could well be the acupuncture meridian which anatomically coincides with connective tissues which also connects to mast cells [38].

Apparently, it is a formidable work to prove and characterize the novel superoxide channels, but what is immediately possible is a real double-blind and placebo-control study of efficacy of acupuncture which is extremely likely to interfere with that novel channels. Hopefully, acupuncture with TAT-SOD but not needle can help accelerate the formation of scientific consensus. One of the major difficulties with clinical study of acupuncture's efficacy is its inevitable placebo effects when a needle is inserted into the body [39], which may contribute to the minor advantages in the efficacy rates reported over our TAT-SOD acupoints treatment.

The enzymatic scavenging of the intracellular superoxide at acupoints in the clinical setting has been demonstrated to achieve an efficacy in treating AR patients comparable to acupuncture, as is evaluated by both clinical measurements and serum biomarkers, proving that our hypothesis that enzymatic scavenging of intracellular superoxide could generate acupuncture-like efficacy is correct. Much more investigation is necessary before a novel regiment of TAT-SOD treatment of AR can be established; the current results clearly suggest a great possibility of replacing a mysterious and cumbersome treatment of acupuncture with an intracellular superoxide scavenging treatment at acupoints no more difficult than applying skin care products.

What is more significant about the confirmation of our hypothesis of this work is that it is true that removal of intracellular superoxide is a key process related to the efficacy of acupuncture treatment. Acupuncture has been proved to be effective, but other than scientifically intangible* Qi* concept and mysterious theory in Traditional Chinese Medicine (TCM), there is no understanding about what exactly happens immediately after a needle is inserted into acupoints in the modern scientific terms, not to mention the whole mechanism of the treatment. On the contrary, it is a matter of fact that TAT-SOD can do nothing but to be delivered into cells to remove superoxide there once it is applied topically to acupoints. Enzymatic removal of intracellular superoxide has generated remarkably similar efficacy in treating AR, suggesting that acupuncture may work on the same mechanism of intracellular removal at acupoints. What is different with acupuncture may be that it removes intracellular superoxide at acupoints by puncturing cells containing superoxide and cause a leakage rather than enzymatic scavenging inside the cells. As is shown by our previous work [14], meridian lines may be the alignment of cells with a high content of superoxide, it is possibly a novel channel for superoxide transporting and disposal between acupoints and the action target of acupuncture, as is in this case a channel between LI 20 and nasal epithelium. It will be a formidable task to answer questions such as where those superoxide anions originate, how they are transported intercellularly along the meridian line, and why they are distributed along the line; nevertheless, the association of meridian system with intracellular superoxide as reconfirmed by this study provides promising new clues to not only the elucidation of the mechanism of effective but overwhelmingly suspected acupuncture treatment but also the discovery of an anatomically adelomorphic system in mammals.

If the intracellular superoxide scavenging at acupoints can be confirmed as a process in the mechanism of acupuncture, enzymatic action by TAT-SOD can provide a convenient solution to the dilemma in the clinical study of acupuncture treatment. The scientific investigation on acupuncture has been suffering from a lack of a reliable placebo in clinical study [39], which fatally jeopardizes the scientific quality of almost all the clinical works [40], contributing to the awkward status of acupuncture not much better than necromantic superstition in spite of being a treatment with the longest history and popularity in human medical history and enormous quantity of researches. TAT-SOD may become a critical tool to upgrade the scientific quality of acupuncture research and accelerate it when further investigations on more conditions confirm the feasibility of substituting acupuncture with TAT-SOD treatment.

## 5. Conclusions

In this study, a novel acupoint therapy of the intracellular superoxide scavenging by TAT-SOD at acupoints was demonstrated to significantly alleviate the symptoms and signs of AR with an overall efficacy comparable to acupuncture, while no improvement was observed with applying the TAT-SOD cream directly to nasal cavity. TAT-SOD treatment may provide a novel and convenient alternative to acupuncture. Moreover, the removal of intracellular superoxide at acupuncture may be an important process in the mechanism of acupuncture.

## Figures and Tables

**Table 1:** Characteristics of subjects in TTA, placebo, and TTN groups, mean (SEM).

Parameter/treatment	TTA	Placebo	TTN	*p* value^*∗*^	*p* value^*∗∗*^
Baseline assessment					
Number	21	17	18	NA	NA
Age (y), mean (SEM)	30.0 (10.3)	30.6 (12.4)	30.2 (11.2)	0.43	0.95
Sex (M/F)	8/13	7/10	7/11	NA	NA
Skin test (number of positive patients)					
Dust mite	18	15	15	NA	NA
Cat	0	0	0	NA	NA
Dog	0	0	0	NA	NA
Mold	3	2	3	NA	NA
Dust mite wheal (mm), mean (SEM)	9.0 (1.2)	9.4 (0.6)	9.2 (0.7)	0.18	0.54
Course of disease (y), mean (SEM)	6.5 (4.8)	7.4 (5.1)	7.5 (3.2)	0.58	0.46
Symptom score, mean (SEM)					
Stuffiness	1.8 (0.6)	1.9 (0.6)	1.8 (0.6)	0.71	0.99
Sneezing	1.4 (0.6)	1.4 (0.6)	1.5 (0.8)	0.89	0.66
Rhinorrhea	1.8 (0.8)	1.9 (0.8)	1.9 (0.6)	0.78	0.67
Itching	1.9 (0.8)	1.9 (0.9)	1.9 (0.8)	0.93	0.99
Total	6.9 (1.8)	7.1 (1.8)	7.1 (1.7)	0.87	0.72
Day 8					
Symptom score, mean (SEM)					
Stuffiness	1.3 (0.6)	1.5 (0.5)	1.6 (0.6)	0.44	0.13
Sneezing	1.2 (0.6)	1.2 (0.5)	1.4 (0.6)	0.93	0.31
Rhinorrhea	1.3 (0.6)	1.8 (1.0)	1.9 (0.6)	0.09	<0.01
Itching	1.3 (0.6)	1.9 (0.6)	1.9 (0.6)	<0.001	<0.001
Total	5.1 (1.3)	6.4 (1.2)	6.8 (1.1)	<0.01	<0.001
Day 15					
Symptom score, mean (SEM)					
Stuffiness	0.8 (0.6)	1.7 (0.6)	1.6 (0.7)	<0.001	<0.001
Sneezing	0.4 (0.6)	1.4 (0.8)	1.4 (0.6)	<0.001	<0.001
Rhinorrhea	1.0 (0.7)	1.5 (0.6)	1.9 (0.6)	0.01	<0.001
Itching	0.8 (0.6)	2.1 (0.7)	2.0 (0.5)	<0.001	<0.001
Total	3.0 (1.7)	6.7 (1.2)	6.9 (1.1)	<0.001	<0.001
Therapeutic response, number (percentage)					
Deterioration	1 (4.8%)	2 (11.7%)	3 (16.7%)	NA	NA
None	3 (14.3%)	14 (82.4%)	15 (83.3%)	NA	NA
Moderate	10 (47.6%)	1 (5.9%)	0 (0%)	NA	NA
Marked	7 (33.3%)	0 (0%)	0 (0%)	NA	NA

NA, not applicable.

^*∗*^Two-sample *t*-test comparing treatment means of TTA versus placebo.

^*∗∗*^Two-sample *t*-test comparing treatment means of TTA versus TTN.
